# Febrile episode unmasking neuropsychiatric systemic lupus erythematosus with lytic lesions caused by secondary autoimmune myelofibrosis: Case report

**DOI:** 10.1097/MD.0000000000028251

**Published:** 2021-12-23

**Authors:** Ionuţ-Flavius Bratu, Athena Cristina Ribigan, Sorina Mihailă-Bâldea, Raluca Badea, Daniela Stefan, Cristina Davidoiu, Bogdan Casaru, Florina Antochi

**Affiliations:** a Department of Neurology, Bucharest Emergency University Hospital, Bucharest, Romania; b Department of Cardiology, Bucharest Emergency University Hospital, Splaiul Independentei, Bucharest, Romania; c “Carol Davila” University of Medicine and Pharmacy, Bucharest, Romania.

**Keywords:** case report, cerebral venous thrombosis, lytic, neuropsychiatric, secondary autoimmune myelofibrosis, systemic lupus erythematosus

## Abstract

**Rationale:**

Systemic lupus erythematosus (SLE) is characterized by numerous immunological abnormalities that lead to multiorgan involvement. Central and peripheral nervous system manifestations are present in 8% to 92% of the cases of SLE. Furthermore, there have been reported cases of secondary autoimmune myelofibrosis associated with SLE.

**Patient concerns:**

We present the case of a 64-year-old female who was transferred from the Cardiology Department, where she was admitted for pericardial-pleural-peritoneal effusion after being discharged from another hospital following the resolution of a febrile episode. During hospitalization, she presented multiple oculomotor nerves palsies and weakness in the lower limbs. Serial cerebral magnetic resonance imaging (MRI) revealed extensive cerebral venous thrombosis. Nerve conduction studies showed sensory-motor axonal polyneuropathy. Thoracic MRI revealed a rare finding in patients with SLE – lytic lesions.

**Diagnoses:**

Extensive clinical, imaging, blood, and urine tests were performed. The patient exhibited pancytopenia, elevated inflammatory markers, hyperhomocysteinemia, mild hypoproteinemia, and severe proteinuria. The Hematology consultation ascertained that the peripheral blood smear and the bone marrow aspiration showed no alterations suggestive for a primary hematological disease and the thoracic vertebral-medullary MRI changes had a very low probability of representing osteolytic lesions in the context of plasma cells dyscrasia, but could not exclude their being result of a secondary autoimmune myelofibrosis. Immunology blood tests highlighted the presence of antinuclear antibodies and lupus anticoagulants. In this context, the Rheumatology consultation established the diagnosis of SLE with multiple complications.

**Interventions:**

The patient received treatment with cyclophosphamide.

**Outcomes:**

The ocular motricity problems and the paraparesis showed improvement. However, 1 week later, the patient developed weakness, dyspnea, and right lower quadrant abdominal pain. The abdominal-pelvic computed tomography scan indicated an acute right retroperitoneal hematoma with active bleeding for which she underwent arterial embolization of the spinal lumbar arteries with optimal result, but she died a few days later.

**Lessons:**

We chose to present this case in order to highlight the importance of interdisciplinarity in diagnosing and managing patients with SLE and multiorgan ailments, especially when faced with rare constellations of complications such as extensive cerebral venous thrombosis and osseous lytic lesions caused by secondary autoimmune myelofibrosis.

## 1 Introduction

Systemic lupus erythematosus (SLE) is characterized by numerous immunological abnormalities that lead to multiorgan involvement. Patients suffering from connective tissue diseases frequently exhibit central and peripheral nervous system complications.^[1]^ The majority of the reported studies highlight that central nervous system (CNS) manifestations are present in 13% to 92% of the cases of SLE, whereas the peripheral nervous system (PNS) manifestations occur only in 8% to 56% of the patients.^[2]^ Furthermore, there have been reported cases of secondary autoimmune myelofibrosis, term used for myelofibrosis associated with a well-defined autoimmune disease (e.g., most commonly SLE, but also dermatomyositis, Sjögren syndrome, or autoimmune hepatitis).^[3]^

We chose to present this case in order to highlight a rare constellation of neurologic and hematological complications of long-standing SLE and its challenges of diagnosis and management. To our knowledge, this is the first case description of a patient with neuro-psychiatric systemic lupus erythematosus (NPSLE) with extensive cerebral venous thrombosis and lytic lesions due to secondary autoimmune myelofibrosis. This case was reported in line with the CARE requirements for case reports.^[4]^

## 2 Case presentation

A 64-year-old female patient with no known medical history or undergoing chronic treatment presented to the Emergency Room of a county hospital for marked asthenia, chills, and fever that had started 2 weeks before. Her blood tests and chest X-ray scan showed the presence of anemia (7.5 g/dL), of an inflammatory syndrome (erythrocyte sedimentation ratio [ESR] – 100 mm/h, C-reactive protein [CRP] – 170 mg/dL), and of radiologic alterations suggestive for interstitial pneumonia and bilateral pleural effusion. She received antibiotic treatment at first with third-generation cephalosporine (for 10 days) and after that with meropenem vancomycin, doxycycline and an antifungal drug. Cardiac and abdominal-pelvic ultrasonography were performed at the time and they revealed the presence of pericardial and pelvic peritoneal effusion. Furthermore, the patient was transferred to the Cardiology Department being suspected of suffering from myopericarditis. During her hospital stay, she experienced an episode of confusion for which she underwent neurological examination, cerebral computed tomography (CT) scan and lumber tap, all of them with normal results. As blood inflammation markers were persistently high and all cultures (cerebrospinal fluid, blood, urine, peritoneal-pleural-pericardial effusion, nasal-pharyngeal) were negative, as well as the PCR SARS-Cov-2 tests, the patient's multiple effusions were suspected of being autoimmune in nature, but she was discharged upon family request against medical advice before further assessment could be performed.

One day later, the patient was admitted to the Cardiology Department of our hospital where she underwent extensive tests in order to determine the etiology of the peritoneal-pleural-pericardial effusion. The main differential diagnosis was based on various type of autoimmune connective tissue diseases, especially SLE and mixed connective tissue disorder. Extensive clinical, imaging, and blood tests were performed. The patient exhibited pancytopenia and elevated CRP and ESR. ECG showed low QRS voltage and negative T wave in the V1-V3 derivations. The cardiac ultrasonography and the blood tests revealed reduced systolic function (left ventricular ejection fraction of 35%) and the presence of anti-nuclear antibodies (more than 1:160). She was diagnosed with myopericarditis with polyserositis, but before further testing could not be done related to the etiology the patient developed neurological signs (left lateral gaze horizontal diplopia with ocular motricity impairment – right eye convergent strabismus and mildly limited abduction of the left eye) and she was transferred to the Neurology Department.

**Figure 1:**
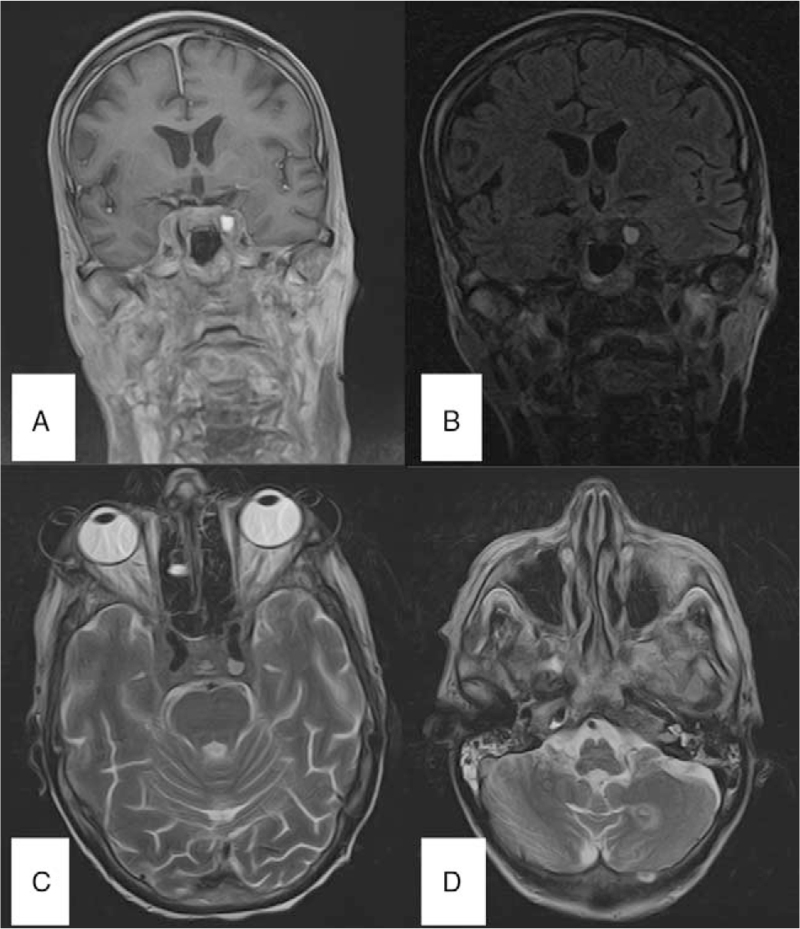
Cerebral magnetic resonance imaging scan – T1-weighted (A), FLAIR (B) coronal sections and T2-weighted (C, D) cross-sections showing 2 non-contrast enhancing T2-weighted/FLAIR hyperintense lesions in the left cavernous sinus and the left cerebellar hemisphere.

Her cerebral CT scan performed at the time was normal. Over the next few days, the patient developed bilateral abducens cranial nerve palsy, multi-direction nystagmus, ageusia, and mild weakness and ataxia of the left upper limb. We performed a cerebral MRI scan that showed no alterations suggestive for meningitis or encephalitis, but it revealed the presence of a noncontrast enhancing lesion in the cerebellum (that could not be distinguished at the time between a vascular or inflammatory lesion) (Fig. 1). Lumbar tap was proposed, but it was postponed due to spontaneously high INR. She was given dexamethasone (8 mg bi-daily) and fresh frozen plasma to lower her INR. As her neurological status declined---she developed bilateral third cranial nerve palsy and paraparesis (4/5 MRC) with absent deep tendon reflexes, a second cerebral MRI scan was performed. It revealed extensive cerebral venous with thrombi present in both cavernous venous sinuses, in the sagittal, transverse, and sigmoid sinuses as well as in the bulb of the right internal jugular vein. She was diagnosed with extensive cerebral venous thrombosis and received enoxaparine. Furthermore, the nerve conduction studies indicated a sensory-motor axonal polyneuropathy of the lower limbs, with mild involvement of the upper limbs for which she received methylprednisolone pulse therapy followed by 48 mg/day of prednisone.

**Figure 2:**
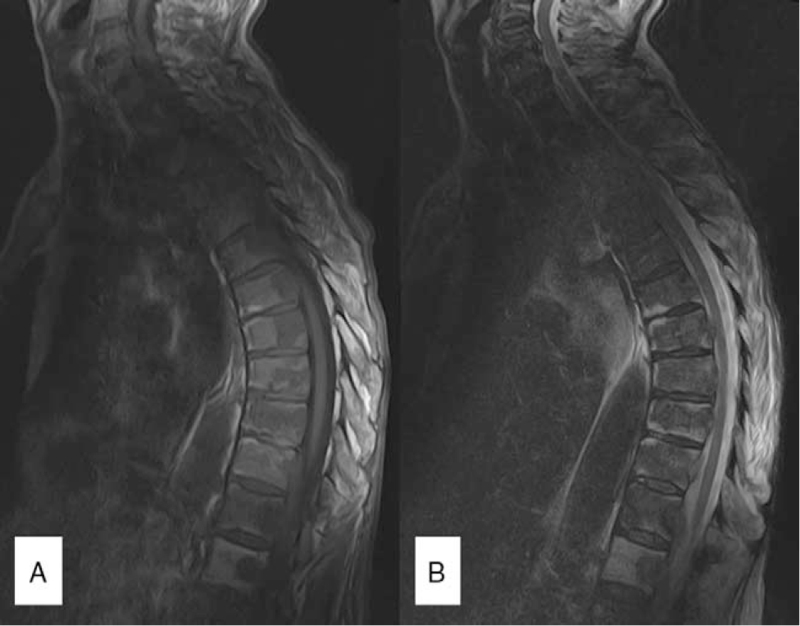
Thoracic magnetic resonance imaging scan – T1 (A) and T2-weighted (B) sagittal sections - thoracic vertebral bodies showing structural, shape alterations, and diffuse signal alterations (T1 and T2 hyposignal). Some of the vertebrae exhibit contrast-enhancement as well as thickening and contrast enhancement of their corresponding leptomeninges.

Extensive clinical, imaging, blood, and urine tests were performed in order to confirm or not the former autoimmune hypothesis. The patient exhibited pancytopenia, elevated CRP and ESR, hyperhomocysteinemia, mild hypoproteinemia (5.45 g/dL), and proteinuria (28.26 mg/dL). The vertebral-medullary MRI scan of the cervical, thoracic, and lumbar segments of the spinal cord showed diffuse alterations in both the structure and the form of thoracic vertebral bodies (Fig. 2). The Hematology consultation ascertained that the peripheral blood smear and the bone marrow aspiration showed no alterations suggestive for a primary hematological disease and the thoracic vertebral-medullary MRI changes had a very low probability of representing osteolytic lesions in the context of plasma cells dyscrasia, but could not exclude their being result of an autoimmune myelofibrosis secondary to a well-defined autoimmune disease. A lumbar tap was performed as well and it revealed normal cellularity and glycorrhachia and slightly raised proteinorrachia (59 mg/dL). Immunology blood tests highlighted the presence of antinuclear antibodies and lupus anticoagulants. In this context, the Rheumatology consultation established the diagnosis of SLE with multiple complications and recommended 800 mg of cyclophosphamide. The ocular motricity problems and the paraparesis showed improvement under this treatment.

One week later, the patient developed generalized weakness, dyspnea, and pain in the right lower quadrant of her abdomen. The blood tests monitoring pinpointed a drop in the value of hemoglobin from 8.1 to 4.3 g/dL over 24 hours. The abdominal-pelvic CT scan indicated an acute right retroperitoneal hematoma with active bleeding. Furthermore, the patient underwent arterial embolization of the spinal lumbar arteries (L1-L3) with optimal post-embolization result and without any periprocedural adverse events. She was transferred to the intensive care unit and underwent surgery, but died a few days later.

## 3 Discussion

In the case of SLE, the neuropsychiatric manifestations can be classified as primary neurological and psychiatric conditions (resulted from direct affliction of the nervous system) and secondary to disease and/or treatment complications.

In terms of the SLE itself being the cause of the neuropsychiatric manifestations, many of them are the result of autoantibody activity: antiphospholipid antibodies (thrombosis, headache, chorea, transverse myelitis, seizures), anti-ribosomal p protein antibodies (psychiatric events), anti NMDA receptor subunits, anti-SM and anti-RNP antibodies (diffuse NPSLE), and anti-AQP4 antibodies (demyelinating lesions).^[5]^ Other incriminated autoantibodies involved in the pathogenesis of NPSLE are anti-endothelial cell, anti-MAP2, and anti-suprabasin antibodies.^[1]^

NPSLE events can be determined by treatment as well: psychosis, infections, and arterial hypertension (cortico-therapy), aseptic meningitis (NSAIDs, azathioprine), and progressive multifocal leukoencephalopathy (cyclophosphamide).^[1]^

Various cerebral imaging aspects were highlighted in patients with NPSLE. The cerebral CT scan shows alterations in 60% of the cases (e.g., cerebral infarction, hemorrhage, abscess, venous thrombosis, white matter anomalies), with cerebral atrophy being the most frequent finding. The cerebral and spinal cord MRI scans can reveal spinal cord lesion, meningeal contrast enhancement, cerebral atrophy, cerebral infarctions, hyperintense periventricular white matter areas and small, punctiform disseminated lesions (which are the most frequent, present in 15–60% of the cases).^[6]^

Current literature does not offer sufficient evidence-based support for consensus and guideline development for treatment of NPSLE. With varying degree of certainty, different studies indicate that there are many therapeutic options at the disposal of the clinician such as high doses of oral corticosteroids, methylprednisolone pulse-therapy, intravenous immunoglobulins (more frequently use in the cases of PNS ailments), plasma exchange, immunosuppressive medication (e.g., cyclophosphamide, azathioprine, mycophenolate mofetil), and biological therapies (e.g., rituximab). Furthermore, hydroxychloroquine was proven to be useful in preventing neurological complications, especially neurovascular event and seizures.^[5]^

Taking into account the troublesome diagnosis pathway, the pathogenesis and the biomarkers for NPSLE an algorithm was proposed by Jeltsch-David and Muller.^[7]^

In SLE, circulating immune complexes and autoantibodies act on the megakaryocyte Fc-receptors causing them to release growth factors that induce collagen production and subsequent myelofibrosis. As there is inefficient hematopoiesis in patients with myelofibrosis, foci of extramedullary hematopoiesis occur, with bone lytic lesions associated with extramedullary hematopoiesis due to myelofibrosis having been reported in cases of SLE.^[8]^

One of the limitations of our case is the lack of a more in-depth nephrological consultation that could ascertain if our patient exhibited secondary membranoproliferative glomerulonephritis in the context of SLE.

From the family perspective, they feel that the outcome was due to the advanced status of her disease and that she received adequate care.

## 4 Conclusion

Interdisciplinarity (neurology-cardiology-rheumatology-radiology-psychiatry-hematology) comes as paramount in diagnosing and managing patients with long-standing SLE and multiple system complications. Even though they are rare, neuropsychiatric problems such as extensive cerebral venous thrombosis and osseous lytic lesions caused by secondary autoimmune myelofibrosis can be encountered in SLE patients and should always bring the diagnosis of SLE into discussion if it had not been put per primam intentionem.

## Author contributions

**Conceptualization:** Ionut Flavius Bratu.

**Data curation:** Ionut Flavius Bratu, Athena Cristina Ribigan, Sorina Mihaila-Baldea, Raluca Stefania Badea, Daniela Stefan, Cristina Rebeca Davidoiu, Bogdan Casaru, Florina Anca Antochi.

**Methodology:** Ionut Flavius Bratu, Florina Anca Antochi.

**Supervision:** Athena Cristina Ribigan, Florina Anca Antochi.

**Validation:** Athena Cristina Ribigan.

**Writing – original draft:** Ionut Flavius Bratu.

**Writing – review & editing:** Ionut Flavius Bratu, Athena Cristina Ribigan, Florina Anca Antochi.
